# Une lesion cutanée persistante non cicatricielle depuis 3 ans pyoderma gangrenosum

**DOI:** 10.11604/pamj.2019.32.198.18692

**Published:** 2019-04-23

**Authors:** Michel Assane Ndour, Djiby Sow, Demba Diedhiou, Fabrice Senghor, Muriel Diembou, Moustapha Diouf, Awa Faye, Ibrahima Mané Diallo, Ahmed Limane Barrage, Léa Marie Kabou, Maimouna Ndour Mbaye, Anna Sarr

**Affiliations:** 1 Service de Médecine Interne, Centre Hospitalier Abass Ndao, Sénégal; 2 Service d’Anatomopathologie, Centre Hospitalier Aristide le Dantec, Sénégal; 3 Service d’Hématologie, Centre Hospitalier Aristide le Dantec, Sénégal; 4 Faculté de Médecine, de Pharmacie et d’Odontologie, Université Cheikh Anta Diop, Dakar, Sénégal

**Keywords:** Pyoderma gangrenosum, dermatose neutrophilique, hyperleucocytose, Pyoderma gangrenosum, neutrophilic dermatosis, leukocytosis

## Abstract

Le pyoderma gangrenosum (PG) est une dermatose neutrophilique non infectieuse rare souvent méconnue. Il se présente généralement par des ulcérations cutanées inflammatoires, très douloureuses et d'évolution rapide. Il est fréquemment retrouvé dans un contexte de néoplasie, de pathologies inflammatoires digestives, rhumatologiques et/ou hématologiques. Son diagnostic est très souvent tardif après de multiples échecs thérapeutiques. Nous rapportons un cas de pyoderma gangrenosum dont le diagnostic n'a pas été criant. Un patient a été admis dans notre service pour une lésion dermatologique persistante et d'évolution défavorable malgré les débridements et l'administration d'antibiotiques. Il était suivi pour un cancer de la prostate, une hypertension artérielle et un asthme. Du fait des anomalies biologiques observées telles qu'une hyperleucocytose à polynucléaires neutrophiles avec myélémie à myélocytes et métamyélocytes, sans blastose sanguine et une anémie normochrome normocytaire, une leucémie myéloïde chronique a été évoquée chez ce patient. Elle a par la suite été infirmée devant les différents examens complémentaires non concluants. C'est ainsi que le diagnostic de PG a été évoqué et confirmé à l'examen anatomopathologique montrant un aspect histopathologique d'un tissu de granulation concordant avec un pyoderma gangrenosum et une absence de signe histologique de malignité. L'institution d'un traitement à base de corticothérapie a abouti à la guérison.

## Abstract (en)

Pyoderma gangrenosum (PG) is a rare non-infectious neutrophilic dermatosis often unknowed. It usually presents with inflammatory skin ulcer, very painful, with rapid evolution. It is commonly found in a context of malignancy, inflammatory bowel disease, rheumatic and/or haematological disease. Its diagnosis is very often late after multiple therapeutic failures. We report a case of pyoderma gangrenosum whose diagnosis was not obvious. A patient was admitted to our department for a persistent dermatological lesion and adverse evolution despite debridements and the administration of antibiotics. He was followed for prostate cancer, high blood pressure and asthma. Due to observed biological abnormalities such as neutrophil leukocytosis with myelocyte and metamyelocyte myeloma, without blood blastosis and normochromic normocytic anemia, chronic myelogenous leukemia was suspected. It was later overturned by the various inconclusive supplementary examinations. This is how the diagnosis of PG was evoked and confirmed by anatomopathological examination showing a histopathological appearance of granulation tissue consistent with pyoderma gangrenosum and no sign of malignancy. The institution of a corticotherapy treatment resulted in the cure.

## Introduction

Le pyoderma gangrenosum (PG) ou ulcère phagédénique est une dermatose bénigne de mécanisme non totalement expliqué appartenant au groupe des dermatoses neutrophiliques. Très douloureux et d'évolution rapide d'allure infectieuse, il contraste avec l'absence d'adénopathie inflammatoire. Son étiologie reste incertaine. Un traumatisme local, même minime peut révéler ou aggraver l'ulcération: c'est le phénomène de Koebner [1]. Le diagnostic est donc souvent tardif, après de multiples traitements antibiotiques et chirurgicaux mutilants avec un grand risque de séquelles fonctionnelles et esthétiques. Il a été mentionné l'existence d'un PG ulcéreux, un PG pustuleux, un PG bulleux et un PG granulomateux superficiel ou végétant [2]. Au travers du cas d'un adulte ayant un Pyoderma gangrenosum ulcéreux persistant, nous rappelons sa présentation clinique et sa prise en charge après une errance diagnostique.

## Patient et observation

**Figure 1:**
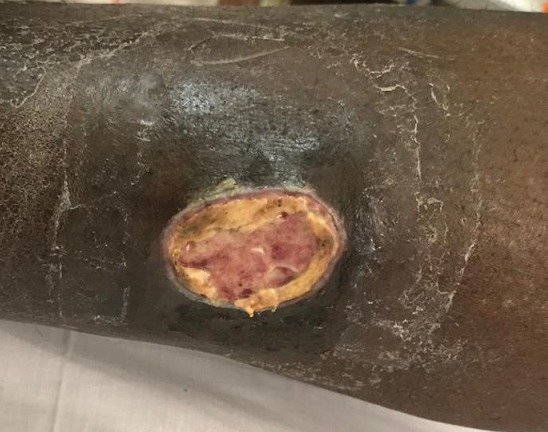
Plaie ulcéreuse à contours hypertrophiques nets et violacés

Monsieur J.C. âgé de 68 ans, a été hospitalisé au Service de Médecine Interne du Centre Hospitalier Abass N'dao à Dakar au Sénégal. La symptomatologie a débuté il y a 3 ans marquée par l'apparition d'une pustule au niveau de la jambe gauche. Dans un but thérapeutique, un débridement a été fait dans une clinique et il a reçu un traitement à base d'antibiotiques pendant un an qui s'est avéré inefficace. Le patient a également eu recourt à la phytothérapie. Malgré ces traitements, il a été observé de nouvelles éruptions de lésions pustuleuses qui s'élargissaient devenant confluentes, et aboutissant à des ulcérations à bords nets, hypertrophiques et violacés (Figure 1). La biologie a montré une hyperleucocytose à polynucléaires neutrophiles à 42.000/mm^3^ avec myélémie à myélocytes et métamyélocytes sans blastose sanguine et une anémie normochrome normocytaire, ce qui a permis d'évoquer une leucémie myéloïde chronique. Cependant les multiples examens complémentaires demandés n'ont pas été convaincants. L'aggravation de son tableau a nécessité une consultation en Médecine Interne. Au cours de son hospitalisation on a constaté une arthrite probablement septique de son genou gauche mais aucun germe n'a été isolé suite à la ponction articulaire. Il a été noté l'apparition d'un séquestre osseux au niveau de la plaie, ce qui a nécessité une séquestrectomie. L'évolution est marquée par un épisode d'instabilité hémodynamique dû à un saignement abondant de la plaie (collapsus) au cours d'un pansement qui a été rapidement pris en charge. Face à l'évolution défavorable en dépit de ces attitudes thérapeutiques, l'absence de germes sur le prélèvement, en plus de l'effet Koebner noté, l'hypothèse de Pyoderma Gangrenosum a été évoquée. Le bilan biologique de contrôle réalisé montrait désormais une hyperleucocytose à 80.000/mm^3^ à polynucléaires neutrophiles. Les coupes histologiques du fragment biopsique montraient un revêtement cutané ulcéré siège d'un tissu de granulation. Il était constitué d'une couche fibrinoleucocytaire, sous-tendu d'un tissu conjonctif lâche richement vascularisé, congestif siège d'un infiltrat inflammatoire polymorphe riche en polynucléaire neutrophile et de quelques foyers de nécrose concordant avec un pyoderma gangrenosum. Notons que le patient était suivi pour un asthme, une tumeur de la prostate et une hypertension artérielle. Le patient a reçu un traitement à base de corticothérapie à raison de 1mg/kg/jour. L'évolution a été favorable marquée par la cicatrisation de la plaie et la régression des paramètres biologiques notamment l'hyperleucocytose.

## Discussion

Chez cet homme de 68 ans, le diagnostic de leucémie myéloïde chronique avait été évoqué à tort avant de retenir celui du pyoderma gangrenosum (PG). Les résultats anatomopathologiques de la pièce de biopsie ont été un infiltrat inflammatoire polymorphe riche en polynucléaire neutrophile et de quelques foyers de nécrose. Le succès de la corticothérapie a affermi cette hypothèse. Le pyoderma gangrenosum est une maladie rare (2 cas/an/million d'habitants) avec une prédominance féminine (76%). L'âge habituel de survenue est compris entre 25 et 55 ans, mais elle peut survenir à tout âge et dès l'enfance [3]. Les lésions prédominent classiquement aux membres inférieurs ou au tronc, mais peuvent s'observer sur l'ensemble de la surface cutanée [4]. Chez notre patient, les lésions étaient localisées au niveau de sa jambe gauche. Les mécanismes physiopathologiques du PG restent pour l'instant très hypothétiques. La nature de l'infiltrat inflammatoire, ainsi que l'anomalie d'expression de divers médiateurs inflammatoires leucocytaires suggèrent que le PG pourrait représenter une dysfonction du système immunitaire ou une éventuelle anomalie des mécanismes inflammatoires qui font suite à une blessure [5]. Dans plus de 50 % des cas, le pyoderma gangrenosum est associé à une maladie systémique [6] sous-jacente à rechercher. Cette dernière peut précéder le PG, mais celui-ci peut aussi la révéler ou la précéder. L'évolution des lésions cutanées est habituellement indépendante de celle de la maladie sous-jacente [7] (tumeurs solides,..). Près de la moitié des cas concernent des patients présentant des pathologies inflammatoires systémiques ou onco-hématologiques. Dans 20 à 60% des cas, les patients atteints de PG présentent une pathologie inflammatoire digestive. Les pathologies rhumatologiques concernent un quart des patients atteints de PG. Il s'agit généralement de la polyarthrite rhumatoïde séropositive, plus rarement de formes séronégatives, de spondylarthropathies ou du lupus érythémateux [8]. Dans le cas que nous avons rapporté, le patient avait une tumeur prostatique et avait développé au cours de son hospitalisation une arthrite aseptique du genou gauche.

Le PG, actuellement intégré au concept de dermatose neutrophilique, peut être confondu ou associé aux autres dermatoses neutrophiliques telles que le syndrome de Sweet (dermatose aigue fébrile neutrophilique), une pustulose sous-cornée ou l'erythema elevatum diutinium [9]. Le diagnostic différentiel des lésions cutanées ulcérées inclut les vasculites, les insuffisances artérielle ou veineuse, les maladies infectieuses et les hémopathies malignes [2]. Chez notre patient, une leucémie myéloïde chronique a été suspectée bien avant son hospitalisation devant des anomalies biologiques. Cette hypothèse a été réfutée devant les différents examens complémentaires non concluants tels que le médullogramme, l'immunophénotypage, la cytogénétique hématologique. À la clinique, le PG se présente initialement par des pustules inflammatoires coalescentes, qui fusionnent progressivement en laissant apparaitre un ulcère nécrotique, avec des bords hypertrophiques bien délimités, de couleur violacée [4] et qui ne guérit pas avec les traitements conventionnels. De nombreux auteurs décrivent un phénomène de pathergie, l'ulcère apparaissant à la suite d'un traumatisme mineur ou à l'emplacement d'un site chirurgical récent [7]. Cette description va parfaitement de pair avec l'observation clinique faite chez notre patient. Le PG connait une évolution rapide et chronique, par poussées intermittentes. Il reste un diagnostic d'exclusion. Les examens biologiques, l'histologie et l'immunofluorescence directe ne sont pas spécifiques et servent plutôt à exclure les diagnostics différentiels [2]. Cependant à l'histologie, on retrouve très souvent un infiltrat inflammatoire à prédominance de polymorphonucléaires neutrophiles, des thrombus localisés ainsi qu'une nécrose de l'épiderme [10] exactement comme dans notre étude. L'absence de germes à l'écouvillonnage de la plaie a fortement conforté notre diagnostic de PG. Dans notre observation, suite à une automédication à long terme, une antibiothérapie et une chirurgie mutilante, une corticothérapie a été efficace avec une cicatrisation de la plaie et une régression des paramètres biologiques dont l'hyperleucocytose. Un traitement d'entretien doit parfois être poursuivi durant plusieurs mois après la régression des lésions. Plusieurs travaux ont également confirmé l'efficacité des anti-TNF tels que l'infliximab [11]. Les soins locaux visent principalement à éviter toute surinfection et la prise en charge d'une éventuelle pathologie inflammatoire ou néoplasique sous-jacente doit évidemment faire partie du plan thérapeutique.

## Conclusion

Le diagnostic de pyoderma gangrenosum doit être évoqué devant tout ulcère nécrotique d'aspect inhabituel, évoluant défavorablement malgré les soins optimaux, associé à une persistance de la neutrophilie. En raison des mécanismes de pathergie, les gestes chirurgicaux devraient être limités au maximum une fois évoqué le diagnostic de PG. Le traitement est basé sur une corticothérapie systémique.

## Conflits d’intérêts

Les auteurs ne déclarent aucun conflit d'intérêts.
